# Retention Royale: a Gamified Territory-Conquest Add-on for Anki to Enhance Engagement with Spaced Repetition

**DOI:** 10.1007/s40670-026-02721-8

**Published:** 2026-04-23

**Authors:** Tobias Whitford, Chase Goldberg, Syed Shehroz Hussain, Mill Etienne

**Affiliations:** School of Medicine, New York Medical College, Valhalla, NY USA

**Keywords:** Medical education, Spaced repetition, Gamification, Anki, Flashcards, Educational technology

## Abstract

Anki is widely used by medical students, yet sustaining motivation remains challenging amid high burnout rates. Retention Royale is a free, open-source Anki add-on that gamifies flashcard review to promote student learning through territory conquest, rewarding study performance with in-game currency. The add-on is available on AnkiWeb (Code: 311670562).

Spaced repetition software has become ubiquitous in medical education, with up to 68.3% of respondents reporting using Anki [1]. Observational studies have demonstrated positive correlations between Anki use and USMLE Step 1 performance, with one study finding that each 1,700 flashcards reviewed was associated with approximately one point increase in score [1]. Despite these benefits, burnout affects approximately 37% of medical students [2], which is associated with reduced motivation and academic engagement. In this context, the delayed gratification inherent in spaced repetition may amplify motivational decline in an already taxed population, compounding adherence challenges.

Gamification, the application of game design elements to non-game contexts, offers a potential solution. A systematic review of 30 randomized controlled trials found that gamification in health professions education was noninferior to traditional techniques and often superior for knowledge acquisition outcomes [3]. Kerfoot and colleagues demonstrated that a competitive online spaced-education game among primary care clinicians improved both knowledge retention and patient outcomes [4]. While gamification add-ons for Anki exist, they have not been well characterized in peer-reviewed literature. Retention Royale is further distinguished by its coin allocation formula, which weights rewards by retention rate rather than card volume. This design reduces the potential for reward through superficial review and positions the system as a pedagogical scaffold rather than an entertainment-focused overlay.

Retention Royale is a free, open-source Anki add-on designed to address this gap by integrating a territory-conquest strategy game with flashcard review. The add-on was developed over 12 months using Python within Anki’s Qt-based plugin framework and is compatible with major operating systems, including Windows, macOS, and Linux.

The core gameplay loop connects review performance to in-game rewards. Upon completing any Anki review session, users earn ‘coins’ calculated as:

$$\mathrm{Coins}=\mathrm{Cards}\;\mathrm{Reviewed}\times\mathrm{Retention}\;\mathrm{Rate}\times10$$

This formula rewards both volume and quality: students who rush through cards with poor recall earn substantially fewer coins than those demonstrating genuine mastery.

Accumulated coins are spent to purchase troops, which are deployed to territories on a map-based interface (Table 1). Users attack adjacent territories through probability-based combat, where casualties scale with the size of the opposing force, and successful conquests provide strategic advantages through regional control bonuses (Fig. 1).

**Table 1:** Alignment of retention royale game mechanics with educational and motivational principles. Interface elements illustrated in Fig. 1, including the territory map, coin balance display, and troop deployment panel, correspond directly to the mechanics described in each row

Game mechanic	Educational or motivational principle	Implementation
Coin formula (Cards × Retention Rate × 10)	Rewards mastery over volume; discourages superficial review	Students with 90% retention earn 1.8× more than those with 50% retention for the same card count
Daily income calculation	Establishes a consistent study routine; links daily effort to tangible progress	Balance updates each session based on that day’s study performance
Territory conquest progression	Goal-setting and achievement motivation	Clear visual progress toward map domination
Real-time multiplayer competition	Social accountability and competitive motivation	Peers compete simultaneously; no turn-based delays
Optional engagement	Preserves learner autonomy; avoids extrinsic motivation crowding out intrinsic motivation	Game layer is fully optional; standard Anki use is unaffected

**Fig. 1:**
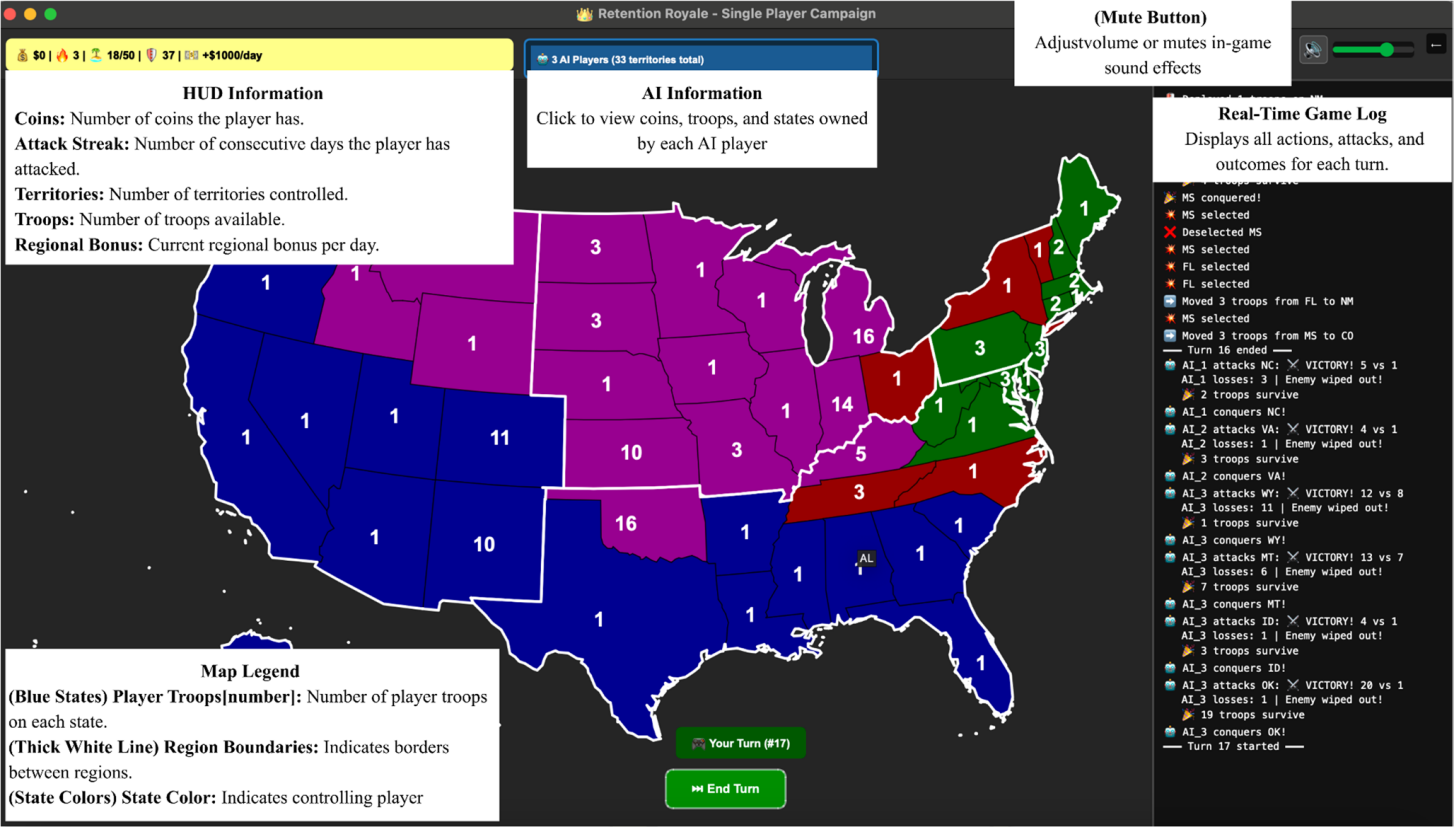
Screenshot of the Retention Royale interface showing the territory conquest map (center), coin balance and troop inventory (upper panel), and game action controls. The color-coded territories correspond to the territory conquest mechanic described in Table 1 (Row 3); the coin counter reflects the performance-weighted earnings formula (Cards Reviewed × Retention Rate × 10) detailed in Row 1; and the troop deployment interface operationalizes the goal-setting mechanic linking study performance to strategic map advancement (Row 3). The optional game panel is overlaid on the standard Anki environment, consistent with the optional engagement design principle described in Row 5

Two game modes are supported. In single-player mode, users compete against one to five artificial intelligence (AI) opponents with adjustable difficulty. Real-time multiplayer lets two to six players compete via shareable game codes, without turn-based delays, enabling continuous interaction. Multiplayer functionality uses Firebase Realtime Database for game state synchronization.

Several design features were implemented to preserve educational integrity. The retention rate multiplier aligns in-game rewards with learning performance rather than solely with review volume. The game layer is optional, allowing users to continue standard Anki use without engaging with Retention Royale. Single-player mode requires no account creation, and multiplayer mode uses anonymous authentication. The add-on does not collect personally identifiable information.

Retention Royale is freely available on AnkiWeb (Add-on Code: 311670562) under open-source license. An integrated tutorial guides users through gameplay mechanics, and a feedback form allows users to report bugs and submit feature requests. Since its release in January 2026, user reviews on AnkiWeb have described the add-on as enhancing motivation and supporting group-based engagement. Formal prospective evaluation of user satisfaction is planned as the next phase of assessment.

This innovation has several limitations that define a clear research agenda. The primary construct targeted is study engagement, specifically sustained adherence to spaced repetition review, rather than immediate performance outcomes. Future investigations should prioritize adherence metrics (daily review completion, session frequency, retention rate trends) before examining downstream examination performance. Early adopters may not represent the broader medical student population, and prospective cohort studies are warranted. The add-on is currently desktop-only; mobile compatibility is a development priority.

Retention Royale represents a novel approach to sustaining medical student engagement with evidence-based spaced repetition learning. By applying territory-conquest gamification mechanics that reward both study quantity and quality, it offers immediate feedback to supplement the delayed gratification inherent in spaced repetition. The add-on is freely available for medical educators and students to implement and adapt.

## Data Availability

Data sharing is not applicable to this article as no datasets were generated or analysed during the current study. The Retention Royale software is freely available on AnkiWeb (Add-on Code: 311670562).
